# Phenotype and pathological significance of MCAM^+^ (CD146^+^) T cell subset in psoriatic arthritis

**DOI:** 10.1007/s11033-021-06678-2

**Published:** 2021-09-07

**Authors:** Smriti K. Raychaudhuri, Christine Abria, Siba P. Raychaudhuri

**Affiliations:** 1 University of California Davis School of Medicine, Sacramento, USA; 2 Sacramento VA Medical Center, 10535 Hospital Way, Mather, CA 95655 USA

**Keywords:** CD146, T-cells, MCAM, Cytokines, Psoriatic arthritis

## Abstract

**Background:**

CD146 (MCAM-melanoma cell adhesion molecule) is a cell surface adhesion molecule for Laminin 411. T cells expressing MCAM are mainly responsible for IL-17 production. IL-17 secreting T helper cells (Th17 cells) are critical for the pathogenesis of psoriatic arthritis (PsA). Here we hypothesized enrichment of CD146^+^IL-17^+^ memory T cells in PsA synovium and studied the association of CD146 expression and CD4^+^IL-17^+^ activated memory (CD11a^+^CD45RO^+^) T cells in synovial fluid and blood of PSA, rheumatoid arthritis (RA, a positive control) and osteoarthritis (OA) patients.

**Methods:**

Hi-D FACS studies were done to identify IL-17 in CD4^+^CD146^+^CD45RO^+^ and CD8^+^CD146^+^CD45RO^+^ T cells.

**Results:**

We observed that effector CD146^+^(MCAM^+^) T cells are enriched at the synovial inflammation site in PsA.

**Conclusion:**

As CD146^+^ T cells are a key resource for IL-17 it is likely that the enrichment of these MCAM^+^ pathologic cells are critical for the disease process of PsA.

## Introduction

Th17 cells play a significant role in various autoimmune and inflammatory diseases [1, 2]. Several recent studies link CD146 (MCAM-melanoma cell adhesion molecule) expression with Th17 effector function. Increased frequency of CD146 expressing Th17 cells has been reported in autoimmune diseases like multiple sclerosis and other forms of inflammatory arthritis [3–5].

CD146 (MCAM) is an immunoglobulin (Ig) superfamily molecule, expressed on endothelial cells, vascular smooth muscle, trophoblast cells and leukocytes. It plays an important role in cell adhesion, tissue invasion and signaling [6, 7]. MCAM expression on lymphocytes was first reported by Pickl et al. [8] as an activation marker of T cells and does not have significant expression on leukocytes of healthy individuals. However, it was found on T cells in synovial fluid of rheumatoid arthritis patients. Circulating human T cells express it ex vivo [9] and is induced by in vitro polyclonal stimulation [8]. Though MCAM is an activation marker of T cells, its distribution on cells is distinct from other activation markers like CD25, CD38, CD69, OX-40, and HLA-DR, [10] there are different degree of MCAM expression with several activation markers [11]. The MCAM^+^ T cells were CD45RA^−^, CD45RO^+^, CD28^+^ and CCR7^−^, like effector memory T cells, and did not have primitive phenotype such as CD133 or CD34 and no endothelial cells associated phenotype [10, 12]. CD26 and CD58 phenotypes associated with Th17 cells were co-expressed on MCAM^+^ cells, while other Th17 cell associated markers, including CD161, CCR6, and CCR4, were partly co-expressed with MCAM [12, 13].

CD146^+^CD4^+^ T cells makes IL-17, IL-22 and (relatively less) IFN-γ [7, 14]. Sixty nine percent of clones of Th17 cells were MCAM^+^ but no Th1 clones was [3]. Peripheral blood CD4^+^CD146^+^ T cells also showed significant intracellular IL-17 and IFN-γ [5, 13] whereas CD4^+^CD146^−^ T cells had little IL-17 secretion but significant IFN-γ production. Supernatants from isolated, CD4^+^CD146^+^ T cells had increased IL-17 compared to CD4^+^CD146^−^ T cells in in-vitro culture. Gene expression analysis also showed increased mRNA for IL17A, IL-22, and IL-26 were higher in CD4^+^CD146^+^ T cells compared to the CD4^+^CD146^−^ T cells, as well as elevated RORC2 [13].

CD146 has significant role in the transendothelial migration of several activated T-helper cell subsets to the inflammation site [14, 15]. Increased CD146^+^ T cells in blood have been reported in autoimmune conditions like RA and other inflammatory arthritides and even higher numbers at the sites of inflammation [11, 13]. There are several reports of MCAM^+^ T cells in multiple sclerosis and suggested their possible role in this inflammatory disease process [3, 16, 17]. In other autoimmune diseases like Sjögren’s syndrome [11] and psoriasis vulgaris [12] increased CD146^+^ T cells were found in peripheral blood. These CD4^+^CD146^+^ T cells were also shown in psoriatic skin lesions and these cells were also producing IL-17 [5] implicating their role in psoriasis in which IL-17 plays a significant role [18–20]. CD146 in the IL-23/IL-17 cytokine network has important role in psoriasis. Here we studied the association of CD146 expression and CD4^+^ IL-17^+^ activated memory T cells psoriatic arthritis (PsA), rheumatoid arthritis (RA) and osteoarthritis (OA) patients’ blood and synovial fluid (SF). CD146^+^ Th17 effector cells have been demonstrated in psoriatic patients’ peripheral blood and skin lesion [5] but not in the synovium of patients with psoriatic arthritis (PsA). Here, we have examined the relationship between CD146^+^CD45RO^+^ memory T cells in respect to their functional significance that is production of IL-17 in PsA patients in a comparative study with PsA, RA and OA.

## Material and methods

### Patient

Peripheral blood from 10 PsA patients [1 female, 9 male; age = 52 years ± 15; mean ± standard deviation (SD)], 10 RA patients [2 female, 8 male; age = 50 years ± 12],10 OA patients (2 female, 8 male; mean age, 55 years ± 12), was obtained. Mean ages were not significantly different between groups (P > 0·05). Patients fulfilled the classification criteria for RA, PsA and OA, had active disease and were not on biologics. Active disease for RA and PsA was defined as morning stiffness > 30 min and > 3 tender/swollen joints. Patients were recruited from Rheumatology/Dermatology clinics at Sacramento VA Medical Center, California, USA. Institutional Review board of Sacramento VA Medical Center approved the study, and all patients gave written informed consent.

### Preparation and stimulation of mononuclear cells

Peripheral blood and synovial fluid mononuclear cells (PBMC and SFMC, respectively) were isolated by density gradient centrifugation using Ficoll-Hypaque (Amersham Pharmacia Biotech, Little Chalfont, UK). Trypan Blue exclusion test showed the cell viability was > 90%.

For cytokine assays, 1 × 10^6^/mL PBMC were cultured in RPMI-1640 medium with 10%v/v heat-inactivated fetal bovine serum, 2 mM glutamine, penicillin/streptomycin and 20 mM HEPES in 24-well culture plates (Nunc, Naperville, IL, USA), coated with anti-human CD3 (Clone UCHT1)(10ug/mL) + soluble CD28 (Clone CD28.2) (2ug/mL) + rIL-23(40 ng/mL) incubated for 3 days. On day 3, the cells were incubated with only rIL-23 (40 ng/mL) for 3 more days. On day 5, phorbol myristate acetate (PMA; 50 ng/mL; Sigma, St Louis, MO, USA), calcium ionomycin (1 μg/mL) (Sigma) GolgiStop monensin at 2·25 μM (Becton Dickinson, Mountain View, CA, USA) were added to cells and incubated for 5 h.

### Immunofluorescent staining and flow cytometry

High definition (Hi-D) multicolor flow cytometry was used to analyze the cell subtypes and intracellular cytokine production of PBMC and SFMC as per our earlier published standardized protocols [21, 22]. The antibodies for surface phenotype staining used were: phycoerythrin (PE)-labelled anti-CD146 (clone P1H12, Millipore), PE-Cy5.5-labelled anti-CD3 (clone OKT3), APC-labelled anti-CD11a (Clone HI111) (Becton Dickinson, Franklin Lakes, NJ, USA), BV421-labelled anti-CD45 RO (Clone UCHL1), APC-Cy7 anti-CD4 (clone RPA-T4), PE-Cy7 anti-CD8 (clone RPA-T8), fluorescein isothiocyanate (FITC)-labelled IL-17 (clone eBio64DEC17) ((eBioscience, San Diego, CA, USA)), aqua labelled Live/Dead cell stain (Invitrogen). Appropriate IgG-conjugated antibodies were used for isotype controls.

First the cells were incubated with antibodies against surface antigens. Then cells were fixed and permeabilized by Perm/Fix solution (Becton Dickinson). Perm/Wash buffer (Becton Dickinson) was used to wash the cells, then stained with intracellular cytokine antibodies and analyzed in FACSAria Fusion analyzer (Becton Dickinson). CD3^+^CD4^+^T lymphocytes or their CD146^+^ and CD146^−^ subsets were gated on live lymphocyte markers. Frequencies of cells expressing surface markers or cytokines of interest were determined using FlowJo™ software (for Windows) Version 10, quadrant statistics (Ashland, OR: Becton, Dickinson and Company 2019).

### Statistical analysis

Prism (version 5·0; GraphPad Software, San Diego, CA, USA) was used for Statistical analyses. Medians and interquartile ranges were used to describe summary statistics. Comparison between groups were done using non-parametric tests (Mann–Whitney U-test or Kruskal–Wallis anova with Dunn's post-test for multiple comparisons).

## Results

### Elevated CD146^+^CD3^+^ T cells in inflammatory arthritis

**Fig. 1:**
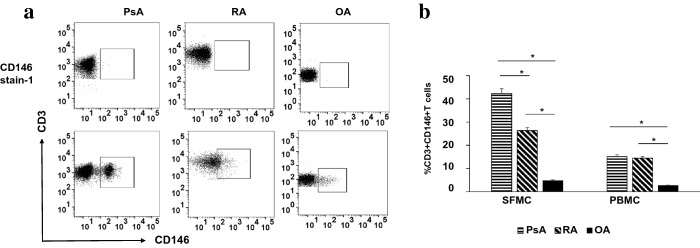
Increased CD146^+^ CD3^+^ T cells in SF and PBMCs of PsA and RA patients: **a** Representative FACS plot from SFMC of psoriatic arthritis (PsA, n = 10), rheumatoid arthritis (RA, n = 10) and osteoarthritis (OA, n = 10) patients. Flow cytometric analysis of CD146^+^ CD3^+^ T cells was done to enumerate the frequencies of these cells in SFMC’s and PBMC’s of PsA, RA and OA subjects. CD146 stain-1 staining was used for control stains for each experiment (**b**). Histogram showing percent positive CD3^+^CD146^+^ cells in PsA, RA and OA of SFMC patients. All experiments were done in triplicate. Results were expressed as mean ± SEM. Mann–Whitney U-test with Dunn's post-test for multiple comparisons were used for comparison between groups. *p < 0.001, SFMC of PsA and RA compared to SFMC of OA and PBMC of PsA and RA compared to PBMC OA

CD146 expression in CD3 T cells was quantified in SFMC’s and PBMCs from PsA (n = 10), patients with RA (n = 10) and patients with OA (n = 10). From within lymphocyte scatter gated cells, CD3^+^ cells were identified and the percentage of CD3^+^ cells expressing CD146^+^ was determined from this subset of T cells. Illustrative examples of CD146 expression in SFMCs are shown in Fig. 1a. We observed that inflammatory arthritis patients had noticeably increased percentage of CD146^+^CD3^+^ T cells than non-inflammatory OA controls (Fig. 1b).

### Increased CD4^+^CD146^+^ and CD8^+^CD146^+^ T cell subsets in synovium of PsA and RA patients

**Fig. 2:**
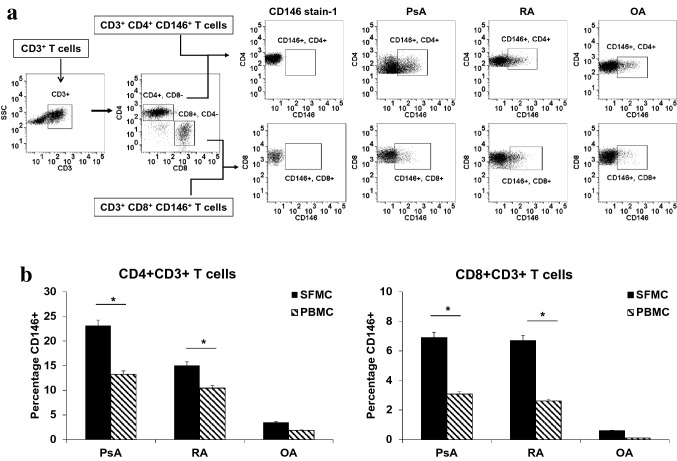
CD4 and CD8 + T cells in SFMC’s of PsA and RA: **a** flow cytometry plots showing gating strategy for identifying CD4 and CD8 T cells expressing CD146. For control staining CD146 stain-1 staining was used for each experiment. Representative plots from SFMC of psoriatic arthritis (PsA, n = 10), rheumatoid arthritis (RA, n = 10) and osteoarthritis (OA, n = 10) patients. **b** Histogram depicting Increase in the frequencies of CD4 and CD8 + CD146 + T cells in SFMC’s and PBMC’s of PsA and RA patients. All experiments were done in triplicate. Results were expressed as mean ± SEM. Mann–Whitney U-test with Dunn's post-test for multiple comparisons were used for comparison between groups. *p < 0.001, SFMC of PsA compared to PBMC of PsA and SFMC of RA compared to PBMC of RA

**Fig. 3:**
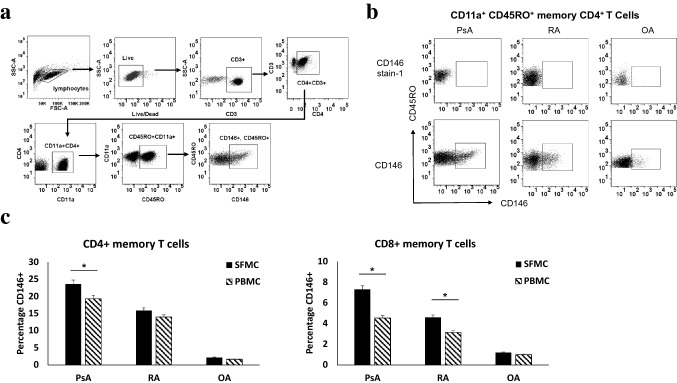
Increased effector memory CD4 and CD8 + T cells in SFMC’s of PsA and RA: **a** Flow cytometry plots showing gating strategy for identifying CD4 memory T cells expressing CD146; the effector memory compartment of T cells as identified by CD11a and CD45RO markers. **b** Representative plots of CD146 expression in CD4 memory T cells of SFMC from PsA, RA and OA patients. **c**. Histograms of CD146 + expression in memory CD4 and CD8 T cells increased in the SFMC compared to peripheral blood of both PsA and RA. All experiments were done in triplicate. Results were expressed as mean ± SEM. Mann–Whitney U-test with Dunn's post-test for multiple comparisons were used for comparison between groups. *p < 0.001, SFMC of PsA compared to PBMC of PsA and SFMC of RA compared to PBMC of RA

Further, we investigated expression of CD146 in CD4^+^ and CD8^+^ T cells. CD4^+^CD146^+^ T cells and CD8^+^CD146^+^ T cells were identified by flow cytometry in the peripheral blood and synovial fluid of PsA, RA and OA patients (Fig. 2a). In PsA SFMC’s, we observed 23.1% ± 3.2 of CD4^+^ and 5.2% ± 0.6 of CD8^+^ T cells expressing CD146; in RA SFMC CD4^+^(17.9% ± 2.6) and CD8^+^ (3.7% ± 0.4) expressed CD146 where as the number for OA were ((CD4^+^(3.5% ± 0.5), CD8^+^0.6% ± 0.5). Compared to OA both in PsA and RA expression of CD146 in both CD4^+^ and CD8^+^ T cell were significantly high (p < 0.01; Fig. 2b). Similar observations were made in the peripheral blood compartments in these three groups (Fig. 2b). Among CD4 compartment, we further evaluated the CD146 expressing effector memory cells on the basis of CD11a and CD45RO co-expression [23] (Fig. 3a,b) and found that the CD146^+^ T cells were enriched up to 23.6% ± 2.7 within memory CD4 T cell compartment of PsA SFMC compared to 2.3% ± 0.2 in OA (p < 0.001) (Fig. 3c).

### IL-17 secretion is differentially enriched in the CD146^+^ memory Th subset

**Fig. 4:**
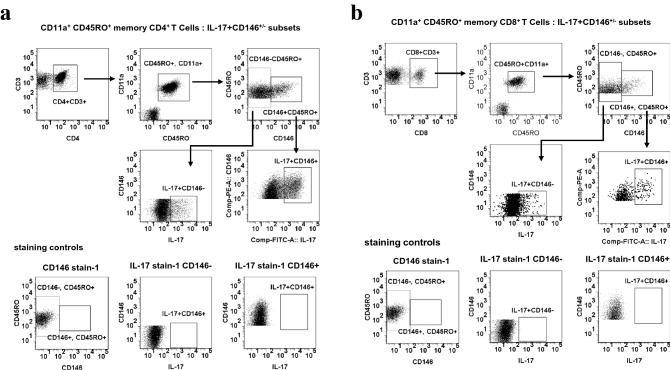
IL-17 secreting cells are enriched in memory CD4 + CD146 + Th cell subset: Flow cytometry plots showing gating strategy to identify **a** CD11a + CD45RO + CD4 + memory T cells with CD146 + and IL-17 + phenotypes and **b** CD11a + CD45RO + CD8 + memory T cells with CD146 + and IL-17 + phenotypes. CD146 stain-1 and IL-17 stain-1 were used as controls for staining in each experiment

To evaluate the relationship of CD146 expression with effector cytokine expression, PBMCs and SFMCs were polyclonally stimulated with CD3/CD28 and recombinant human IL-23, as described in method section. In each group, a majority of CD146^+^ PBMCs, and almost all IL-17^+^ lymphocytes, were within the CD4^+^ subpopulation; a representative illustration from the same PsA patient is shown in Fig. 4a. Secretion of IL-17 was readily detectable. The median frequency of IL-17-secreting CD4^+^ and CD8^+^ memory T cells among CD146^+^ was approximately (26.7% ± 2.1 and 6.53% ± 1.6 respectively) compared with CD146^−^ cells (p < 0.05) (Fig. 4b).

**Fig. 5:**
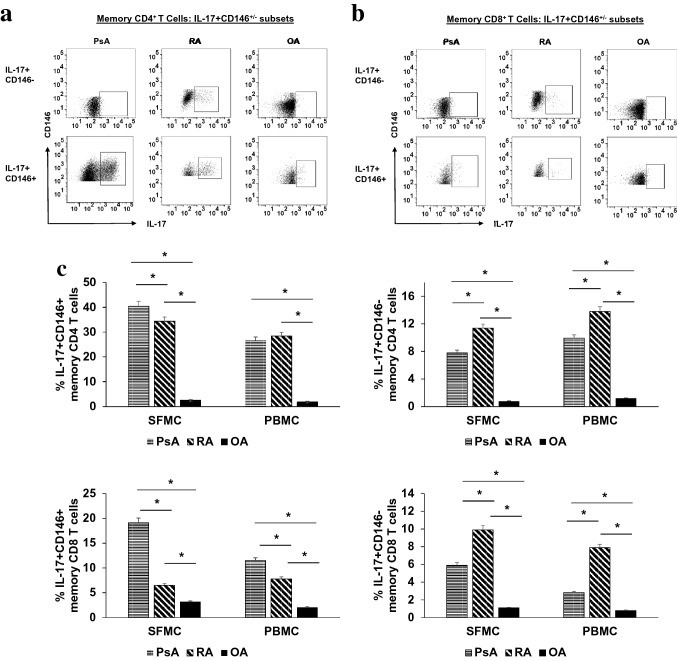
IL-17 secreting cells are enriched in memory CD4 + CD146 + Th cell subset: **a** Representative plots of IL-17 expression in CD146(+ and −) cells within CD11a + CD45RO + CD4 + memory T cells of SFMC from PsA (n = 10), RA (n = 10) and OA (n = 10) patients. **b** Representative plots of IL-17 expression in CD146(+ and −) cell within CD11a + CD45RO + CD8 + memory T cells of SFMC from PsA, RA and OA patients. **c** Histogram showing percent of IL-17 + cells in CD146 + or CD146- memory CD4 and CD8 cells in SFMC and PBMC of PsA, RA and OA. All experiments were done in triplicate. Results were expressed as mean ± SEM. Mann–Whitney U-test with Dunn's post-test for multiple comparisons were used for comparison between groups. *p < 0.001, SFMC of PsA and RA compared to SFMC of OA in. *p < 0.01 SFMC of PsA and RA compared to SFMC of OA and PBMC of PsA and RA compared to PBMC of OA.

FACS studies revealed that effector cytokines were secreted by both CD146^+^ and CD146^−^ memory T cells (Fig. 4a). However, a higher proportion of CD146^+^ than CD146^−^ T cells (CD4^+^ or CD8^+^) were IL-17^+^ (Fig. 5a,b). The enrichment of IL-17 producers in the CD146^+^ population was (twofold increase for both memory CD4 & CD8 PBMC; fivefold increase in memory CD4 SFMC, fourfold increase in memory CD8 SFMC) fold (Fig. 5c). The CD146^+^ IL-17 secreting memory CD4 and CD8 T cells were significantly increased among PsA and RA synovial fluid and PBMC’s in comparison to that of OA (p < 0.001). However, we observed that CD11a^+^CD45RO^+^ CD146^+^CD4^+^ IL-17^+^ memory T Cells are significantly more enriched compared to CD11a^+^CD45RO^+^ CD146^+^CD8^+^ IL-17^+^ memory T Cells at the synovial inflammation site in PsA and RA (p < 0.01).

## Discussion

Given an important role of the CD146 in the IL-23/IL-17 cytokine network and in psoriasis we studied the association of CD146 expression and CD4^+^ IL-17^+^ activated memory T cells in PsA, RA and OA patients, in the blood as well as in the synovial fluid (SF). CD146^+^ T cells have been suggested as a new pro-inflammatory subset of T cells. CD146^+^ T cells are relatively new in the field of human autoimmune diseases. Earlier CD146^+^ T cells T cells have identified in inflammatory arthritis such as in RA, PsA and reactive arthritis [4, 24] but functional significance of the effector phenotype that is activated memory (CD146^+^ CD45RO^+^) T cells have not been elucidated. Here aim of our study was to delineate in detail CD146^+^ T cell subpopulation phenotype in PsA in respect to their effector T cell function and enrichment in the synovium. To our knowledge this is the first report about the functional significance of activated CD4^+^CD146^+^CD45RO^+^ and CD8^+^CD146^+^ CD45RO^+^ T cells in PsA. In this study, we analyzed the relationship between IL-17 production (following short polyclonal stimulation) and CD146 expression in total CD3/CD4/CD8 in the activated naive and memory T cells ex vivo in PsA and RA patients as well as in OA patients as an inflammatory and a non-inflammatory arthritis control.

The frequency of circulating CD146^+^ T cells was increased in PsA and RA patients compared to OA (Fig. 1a, b). CD146^+^ T cells were enriched even further in synovial fluid of PsA and RA patients in comparison to OA. PsA patients had significantly higher CD4^+^CD146^+^ T cells in PBMC compared to OA (13.25 ± 3.41 vs 1.85 ± 0.4 respectively, p < 0.01, Fig. 2b). Also, SF of PsA patients showed a significant higher level of CD4^+^CD146^+^ T cells compared to OA (23.1% ± 3.2 vs 3.5% ± 0.5; p < 0.01, Fig. 2b) compared to PBMC. Similar results were seen in RA patients also (Fig. 2b).

Almost all CD146^+^ CD4/CD8 T and cells were CD45RO^+^ memory phenotype, whereas CD146 negative CD4/CD8 cells as expected included both memory and naive populations. Synovial enrichment of CD146^+^ memory T cells (both CD4^+^ and CD8^+^) at the site of pathogenesis in comparison to blood demonstrating CD146-dependent recruitment to sites of inflammation and further its correlation with IL-17A cytokine secretion (Fig. 5c) provide evidence of critical role of CD146 in the proliferative and inflammatory cascades of PsA. The greater enrichments observed in SF for IL-17 may be due to co-expression of CD146 and this effector cytokine. Moreover, we confirmed recent observations that a subset of RA patients has elevated frequencies of circulating CD146^+^CD4^+^ T cells. Together, these findings are consistent with our proposed hypothesis that CD146^+^, are enriched at the inflamed joints and CD146 likely to be a key regulator for the Th17 effector function in psoriatic disease. These observations indicate a relationship between Th17 effector function of CD4 and CD8 memory T cells and CD146 expression in inflammatory arthritis.

## Conclusion

In conclusion, the current data demonstrate that CD4^+^CD146^+^ activated memory T cells (CD45RO^+^) lymphocytes preferentially accrue in synovial fluids and express distinct phenotypes from the CD146^−^ (negative) memory T cells (Fig. 4a). These observations suggest that circulating CD146^+^ T cells may represent a group of cells with both the means to extravasate to the site of inflammation (via CD146 expression), and to mediate inflammation at a specific site. As CD146^+^ T cells are the key source of the lesional Th17 cytokines it is likely enrichment of these CD146^+^ pathologic T cells plays a significant role in PsA disease process. It is important to mention that in PsA the major source of IL-17A are the activated CD4^+^CD45RO^+^ effector memory T cell [25, 26]. CD146^+^ subset of CD4^+^ T cells in addition to IL-17 also makes IL-22 and (to a lesser extent) IFN-γ [7, 14]. Thus, these CD4^+^CD45RO^+^ T cells expressing MCAM (CD146) are distinctively capable of secreting multiple cytokines responsible for psoriatic pathogenesis. This unlocks a new avenue for therapy for psoriasis and PsA. MCAM can be a target molecule for multiple crucial cytokines of psoriatic disease such as IL-17, IL-22 and IFN-γ and thus may be more effective. Instead of targeting individual cytokines, which often have redundant effects, targeting MCAM may provide the possibility of the inhibiting of multiple cytokines.

## Data Availability

The datasets generated during and/or analyzed during the current study are available from the corresponding author on reasonable request.
